# Introduction of the second-generation direct-acting antivirals (DAAs) in chronic hepatitis C: a register-based study in Sweden

**DOI:** 10.1007/s00228-018-2456-y

**Published:** 2018-04-09

**Authors:** P. Frisk, K. Aggefors, T. Cars, N. Feltelius, S. A. Loov, B. Wettermark, O. Weiland

**Affiliations:** 1 Public Healthcare Services Committee, Stockholm County Council, P.O. Box 6909, S-102 39 Stockholm, Sweden; 2 Department of Pharmaceutical Biosciences, Uppsala University, Uppsala, Sweden; 3 Department of Medical Sciences, Uppsala University, Uppsala, Sweden; 4 Medical Products Agency, Uppsala, Sweden; 5 Department of Medicine Solna, Clinical Epidemiology Unit/Clin. Pharmacol, Karolinska Institutet, Stockholm, Sweden; 6 Department of Medicine, Division of Infectious Diseases, Karolinska Institutet at Karolinska University Hospital Huddinge, Stockholm, Sweden

**Keywords:** Hepatitis C, Direct-acting antivirals, Effectiveness, Managed introduction

## Abstract

**Purpose:**

Introduction of the direct-acting antivirals (DAAs) for treatment of chronic hepatitis C (CHC) infection has been challenging in all health systems. In Sweden, a national protocol for managed introduction was developed. It was optional, but all county councils agreed to implement and follow it. The purpose of this study was to study (a) cure rates among all patients initiated on treatment in 2014–2015, (b) prescribers’ adherence to the drug recommendations and treatment eligibility criteria in the protocol, and (c) introduction rate in the six Swedish healthcare regions.

**Method:**

A cross-sectional study where national data from the Prescribed Drug Register and the quality register InfCare Hepatitis defined the study population, and clinical data from the Patient Register and InfCare Hepatitis were used to monitor outcomes. Descriptive statistics were used.

**Results:**

A total of 3447 patients were initiated on treatment during 2014–2015. The overall cure rate, based on data from 85% of the cohort, was 96%, with variation between genotypes. Adherence to drug recommendations increased over time and varied between 43.2 and 94.2%. Adherence to the treatment eligibility criteria was initially 80% and increased to 87% when treatment restrictions were widened. The introduction rate differed initially between the regions and reached stable levels 15–18 months after the launch of the first DAA.

**Conclusion:**

The estimated overall cure rate was 96%, with some variations between genotypes. A high level of adherence to the introduction protocol as well as similar introduction rates in the health care regions indicate that the introduction protocol, alongside with other measures taken, contributed considerably to a rapid uptake and equal distribution of DAAs in Sweden.

## Introduction

A majority of patients who become infected with hepatitis C virus (HCV) develop chronic hepatitis C (CHC). Left untreated, fibrosis in CHC will after on average 30 years have progressed to liver cirrhosis in a third of the patients, and many will develop hepatocellular cancer [1]. CHC is estimated to affect some 170 million people worldwide [1], with a rising incidence and prevalence [2]. Currently, the worldwide prevalence is estimated to approximately 2.8% [2]. Considering the consequences for both individuals and society, effective and affordable treatment options are urgently needed.

Drug treatment for chronic hepatitis C infection has improved markedly in recent years. The addition of first-generation protease inhibitors (PI), telaprevir or boceprevir, to pegIFN-α and ribavirin resulted in substantially increased cure rates for patients with HCV genotype 1 [3]. Addition of the first-generation PIs was however associated with many severe side effects and more complex drug-drug interactions than pegIFN and ribavirin treatment alone [4]. The adverse events associated with pegIFN and ribavirin therapies remained, and were enhanced with triple therapy including first-generation PIs. Furthermore, an additional pill-burden resulted in problems with poor adherence [4]. Clinical trial data with new direct-acting antiviral agents (DAAs) launched in 2014, however, showed high cure rates and few side effects with shorter treatment durations. Effective antiviral treatment for most genotypes had thus become available, and for the first time, IFN-free treatment options could be offered [5].

In Sweden, access to healthcare is provided by 21 county councils (autonomous healthcare regions), and is tax funded [6]. The right of licensed physicians to unrestricted prescribing and the fact that each county council is responsible by law for its own drug budget constitute the legal framework for prescribing drugs in Sweden. Prescription drug costs are largely covered by each county council, which in turn receive annual reimbursement from the government. A national scheme regulates patient co-payment [7]. Patients with known CHC infection are mainly cared for as outpatients by hospital-based specialists in infectious diseases or gastroenterology/hepatology. To promote efficient disease control, the cost of drugs intended for certain infectious diseases including hepatitis C are by the communicable disease law free of charge to the patient [8]. In a recent Swedish register-based study, the overall prevalence of CHC infection and treated disease are estimated to be 0.36 and 0.01%, respectively [9].

**Fig. 1:**
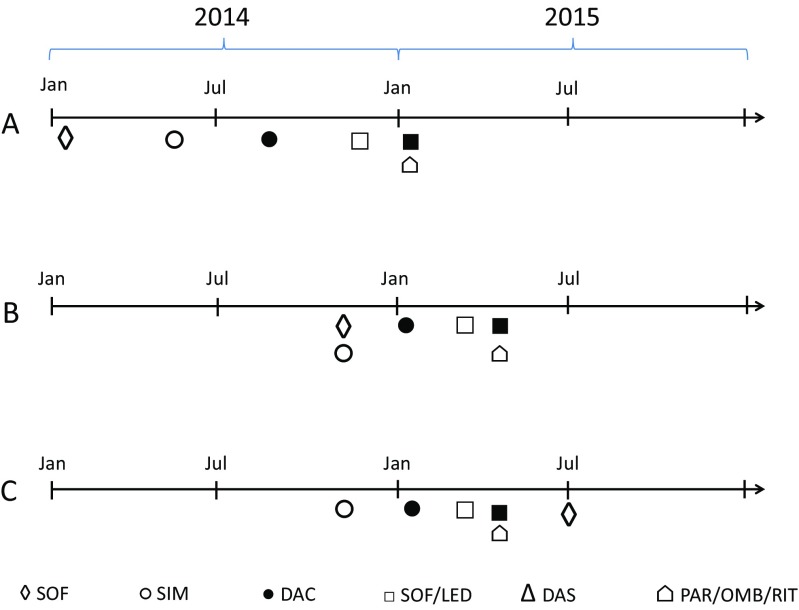
Timelines for regulatory approval (**A**), inclusion of drug in introduction protocol (**B**), and risk sharing agreement (**C**). SOF, sofosbuvir; SIM, simeprevir; DAC, daclatasvir; SOF/LED, sofosbuvir/ledipasvir; DAS, dasabuvir; PAR/OMB/RIT, paritaprevir/ombitaprevir/ritonavir

Soon after the introduction of sofosbuvir and simeprevir (Fig. 1), the Medical Product Agency and their reference group of specialists on antiviral therapy published treatment guidelines based on available clinical evidence [10]. Since the new DAAs were high-priced, easier to use and very promising regarding effectiveness, a considerable increase in expenditure and potentially large regional differences in uptake was anticipated. Therefore, a national introduction process, coordinated by the Swedish Association of Local Authorities and Regions (SALAR) and involving county councils, the Medicinal Products Agency, The Dental and Pharmaceutical Benefits Agency and specialists, was initiated in early 2014, as a pilot project guided by the new working process for the managed introduction of new drugs [11]. It resulted in an introduction protocol issued by the New Therapies Council, an expert group supporting the county councils. The protocol contained clinical background information on currently and soon to be available DAAs, and guidelines on how to prioritize patients for treatment with DAAs and on which drugs to choose. The first protocol with guidelines based on both clinical recommendations and health economic assessments was implemented on November 7th 2014, about 9 months after the regulatory approval of sofosbuvir (Fig. 1). Even though it was not mandatory from a policy perspective, the protocol was accepted by all involved actors and updated repeatedly when new drugs were launched and received a health economic assessment. Simultaneously, price negotiations between all county councils, The Dental and Pharmaceutical Benefits Agency and the pharmaceutical companies took place, resulting in risk sharing agreements. Over time, these were included in the introduction protocols since they—along with the available clinical evidence and health economic assessments—had an impact on which drugs were recommended (Table 1) [12]. Only prescribers specialized in treating patients with CHC were allowed to prescribe. Initially, treatment was restricted to patients with advanced fibrosis/cirrhosis (fibrosis stage 3 and 4). This restriction was relieved to also include patients with moderate fibrosis (fibrosis stage 2) from July 2nd, 2015 (Table 2). In addition, there were regional variations in other cost-containment strategies, with the southern region implementing a patient-level prior authorization scheme and the middle, northern, western, and south-east regions implementing different regional approval procedures, where clinicians could nominate new therapies for approval on a county or regional level.

**Table 1:** First line treatment with DAAs by genotype (Gt), version of introduction protocol and stage of fibrosis (F)

	2014-11-07 (2.0)	2015-01-12 (3.0)	2015-03-06 (4.0)	2015-04-13 (5.0)	2015-07-02 (6.0)
Gt 1	F3–F4 SOF + SIM	F3–F4 Treatment naïve: SOF + SIM Previously treated: SOF + SIM or SOF + DAC	F3–F4 SOF/LED	F3–F4 SOF/LED or OMB/PAR/RIT + DAS	F3–F4 SOF/LED or OMB/PAR/RIT + DAS F2 SOF/LED
Gt 2	F3–F4 SOF + ribavirin	F3–F4 SOF + ribavirin	F3–F4 SOF + ribavirin	F3–F4 SOF + ribavirin	F2–F4 SOF + ribavirin
Gt 3	F3–F4	F3–F4 SOF + DAC	F3–F4 SOF + DAC or SOF/LED	F3–F4 SOF + DAC or SOF/LED	F3–F4 SOF + DAC or SOF + ribavirin F2 SOF + ribavirin
Gt 4	F3–F4 SOF + SIM	F3–F4 Treatment naïve: SOF + SIM Previously treated: SOF + SIM or SOF + DAC	F3–F4 SOF/LED	F3–F4 OMB/PAR/RIT + ribavirin	F3–F4 SOF/LED or OMB/PAR/RIT + ribavirin F2 SOF/LED

Ribavirin could be added where suitable. Treatment duration (varying with disease severity) is not presented here. Previous treatment status refers to previous use of telaprevir and boceprevir

*SOF* sofosbuvir, *SIM* simeprevir, *DAC* daclatasvir, *SOF/LED* sofosbuvir/ledipasvir, *DAS* dasabuvir, *PAR/OMB/RIT* paritaprevir/ombitasvir/ritonavir

**Table 2:** Treatment eligibility criteria

	Conditions	Data source(s)	Measurements
Treatment eligibility (before July 2nd 2015)	Stage of fibrosis 3–4 and/or cirrhosis	InfCare Hepatitis	Measured with the Batts-Ludwig or Metavir scale or the corresponding disease stage measured with other rating scales [27]. Verified by Fibroscan measurement ≥ 9.4 kPa, APRI-score > 2, and/or clinical diagnosis
	Transplantation at any time before treatment initiation	National Inpatient Register 1997–2015	ICD10-codes Z94.0 (renal transplant), Z94.1 (heart transplant), Z94.2 (lung transplant), Z94.3 (heart and lung transplant), and Z94.4 (liver transplant)
Treatment eligibility (from July 2nd 2015)	Stage of fibrosis 2–4 and/or cirrhosis	InfCare Hepatitis	Measured with the Batts-Ludwig or Metavir scale or the corresponding disease stage measured with other rating scales [27]. Verified by Fibroscan measurement ≥ 7.0 kPa, APRI-score > 2, and/or clinical diagnosis
	Transplantation at any time before treatment initiation	National Inpatient Register 1997–2015	ICD10 codes Z94.0 (renal transplant), Z94.1 (heart transplant), Z94.2 (lung transplant), Z94.3 (heart and lung transplant), and Z94.4 (liver transplant)

Our aims were to study treatment outcome in all patients starting treatment with DAAs during 2014–2015 and to study prescribers’ adherence to the drug recommendations and treatment eligibility criteria presented in the introduction protocol implemented in Sweden in 2014–2015, covering the introduction of sofosbuvir, simeprevir, daclatasvir, ledipasvir/sofosbuvir, dasabuvir, and paritaprevir/ombitasvir/ritonavir for the treatment of CHC. Furthermore, the rate of introduction in the different health care regions was studied.

## Methods

### Data sources

This was a cross-sectional study, with registry data covering the whole country. The data sources used were the Swedish Prescribed Drug Register [13] and the Swedish National Patient Register [14], both held by the National Board of Health and Welfare, and the national quality register InfCare Hepatitis, held by the Karolinska University Hospital, Stockholm. The Swedish Prescribed Drug Register is a mandatory national register with information about each individual and their prescription drug purchases (age, sex, Anatomical Therapeutic Chemical [ATC] codes, prescribing and dispensing dates, number of packages, and prescribed dose). The National Patient Register is also a mandatory national register, with individual level data on documented main and contributory diagnoses (ICD codes) and diagnostic and clinical measures taken. It contains data on all hospitalizations and consultations to specialists in ambulatory care, but not consultations in primary care. InfCare Hepatitis is a national database including clinical data from 87% of the infectious disease clinics in Sweden, and one of the gastroenterology clinics. It was started in 2009 and serves primarily as a clinical decision support and benchmarking tool, but can also be used for research [12]. In this study, InfCare Hepatitis is considered a treatment-specific quality register, not disease specific, as the current number of untreated patients in the register is low [15].

Data from these sources were linked on an individual level, using the personal identification number (PIN) [16].

### Study population

Patients who were dispensed or administered any of the following drugs in Sweden during 2014–2015 and who therefore are detected in either the Swedish Prescribed Drug Register and InfCare Hepatitis or in the Prescribed Drug Register only were included: sofosbuvir (ATC code J05AX15), simeprevir (ATC code J05AE14), daclatasvir (ATC code J05AX14), sofosbuvir/ledipasvir (ATC code J05AX65), dasabuvir (ATC code J05AX16), and ombitasvir/paritaprevir/ritonavir (ATC code J05AX67).

### Definitions

The analysis of treatment outcome was based on total treatment given, including potential add-on treatment given when the patient did not respond sufficiently to the initial treatment and re-treatment for patients with a relapse.

Adherence to drug recommendations was defined as initiation of treatment with the drug or drugs recommended for the genotype in question at the time of treatment initiation. This analysis includes all patients with genotype 1–4 starting treatment from November 7th 2014, when the introduction protocol was implemented. Time of treatment initiation is based on the date of first dispensing in the Prescribed Drug Register or the treatment start date in InfCare Hepatitis (for patients without data in the Prescribed Drug Register).

The treatment eligibility criteria before and after July 2nd, 2015 respectively, were based on stage of fibrosis, prevalence of cirrhosis, and/or previous transplantation (Table 2). Adherence to the treatment eligibility criteria was defined as initiation of treatment in a patient who, at the time of initiation, did fulfill at least one of the criteria.

### Outcome measures

Treatment outcome, i.e. cure rate, was measured as the proportion of patients per genotype who achieved a sustained viral response, i.e. undetectable levels of HCV-RNA in plasma 12 weeks after end of treatment (SVR12).

Adherence to the introduction protocol was measured as follows:

- the proportion of patients who were treated with the drug or drugs recommended as first-line treatment for their specific genotype at the time of treatment initiation and
- the proportion of patients who met the treatment eligibility criteria before and after July 2nd, 2015, at the time of treatment initiation.

Introduction rate was measured as the relative proportion of patients on treatment per region over time, and comparisons were made between the six healthcare regions: northern, middle, south-east, western and southern Sweden, and the region of Stockholm-Gotland, respectively. The general population distribution of the regions was based on population statistics dated June 30th 2015, from Statistics Sweden.

InfCare completeness was calculated as the number of patients with the specific characteristic in InfCare divided by  the number of patients in the study cohort.

### Statistics

Descriptive statistics, such as numbers and proportions, were used to describe the study cohorts and the utilization patterns, with 95% confidence intervals (95% CI) where appropriate. Analyses were stratified by sex and age in the categories 0–20, 21-30, 31-40, 41-50, 51-60, 61-70, 71-80 and ≥ 81 years. Means were presented with standard deviations (SD). Data were analyzed in SAS Enterprise Guide 6.1. (SAS Institute, Cary, NC).

### Ethics

The study was reviewed and approved by the Regional Ethical Review Board in Stockholm, approval no. 2015/497-31/1.

## Results

The cohort of patients initiated on the drugs included consisted of 3447 patients. In a majority of patients, clinical data relevant for the aims of this study were available (Table 3).

**Table 3:** Characteristics of patient population

No. of patients	3447	% of cohort with missing data
Age (years), mean (SD)	55.8 (10.8)	0
0–20	10 (0.3%)	
21–30	95 (2.8%)	
31–40	250 (7.3%)	
41–50	544 (15.8%)	
51–60	1404 (40.7%)	
61–70	957 (27.8%)	
71–80	179 (5.2%)	
81–	8 (0.2%)	
Proportion women	33.5%	
Genotype (*n* = 3016)		12.5%
1	1708 (56.6%)	
2	317 (10.5%)	
3	870 (28.8%)	
4	112 (3.7%)	
5	2 (0.1%)	
6	7 (0.2%)	
Estimated stage of fibrosis at treatment initiation* (*n* = 2672)		22.5%
F0–F1	319 (11.9%)	
F2	473 (17.7%)	
F3	588 (22.0%)	
F4	1292 (48.4%)	
Previous transplantation	266 (7.7%)	

*Registered stage of fibrosis in InfCare Hepatitis is often based on liver biopsy. Where a registered stage of fibrosis was missing; an estimated value was assigned based on measurement of liver elasticity (Fibroscan) or APRI-score, if possible

The analysis of treatment outcome included all patients with documented viral load measurements 12 weeks after the end of treatment (2921 patients, 85% of the cohort). Sustained viral response was achieved in 96.1% of the patients, with some variation between genotypes (Table 4).

**Table 4:** Cure rates per genotype

Genotype	No. of patients	Proportion with SVR12 (± 95%CI)
1	1631	97.6 ± 0.8
2	289	95.2 ± 2.5
3	788	93.8 ± 1.7
4	107	97.2 ± 3.1
5	2	100.0
6	7	100.0
Genotype missing	97	92.8 ± 5.2
Total	2921	96.1 ± 0.7

The analysis of adherence to drug recommendations includes all patients with genotype 1–4 treated from November 7th 2014 (2253 patients, 65% of the cohort). On an overall level, adherence to drug recommendations was initially moderate, 43.2% (95% CI 37.5–48.9%), and increased over time to 94.2% (95% CI 92.7–95.6%), for treatment initiation done after July 2nd 2015. Adherence varied considerably between genotypes (Table 5).

**Table 5:** Adherence to drug recommendations over time

Genotype	Protocol version (proportion with prescribing as recommended, ± 95% CI)					
	2.0	3.0	4.0	5.0	6.0	*n*
1	57.6 ± 7.6%	79.8 ± 5.6%	72.7 ± 7.4%	94.0 ± 3.2%	94.3 ± 1.9%	1302
2	92.6 ± 9.9%	85.4 ± 10.9%	84.2 ± 16.4%	92.3 ± 10.3%	82.8 ± 7.5%	212
3	0%	91.7 ± 5.0%	96.7 ± 4.5%	96.3 ± 3.6%	97.9 ± 3.4%	663
4	66.7 ± 37.8%	88.9 ± 14.5%	85.7 ± 26.4%	15.4 ± 19.6%	93.8 ± 8.4%	76

Includes all patients with documented genotype 1–4, starting treatment as of November 7th, 2014 (2253 patients, 65% of population)

**Fig. 2:**
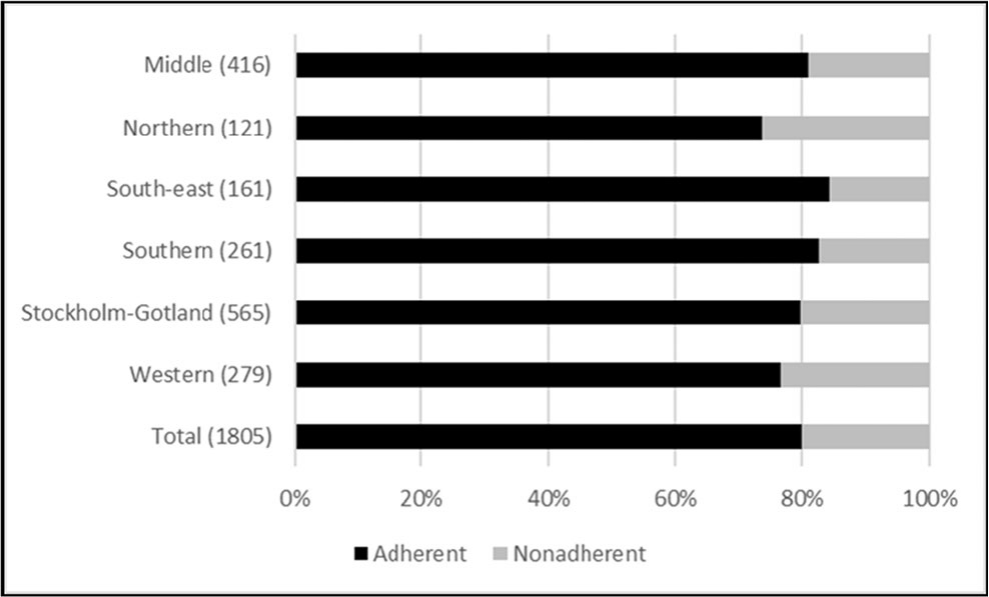
Prescribers’ adherence to treatment eligibility criteria effective before July 2nd 2015, presented by healthcare region. Data representing two patients with missing information on county of residence are omitted from the region columns, but included in total. The number of patients per region is presented within brackets

**Fig. 3:**
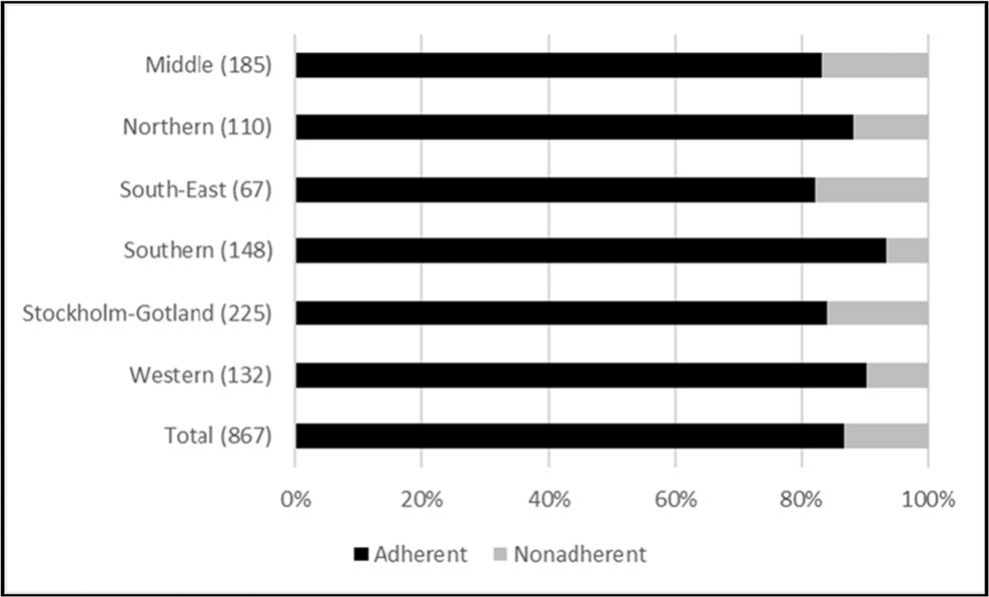
Prescribers’ adherence to treatment eligibility criteria effective from July 2nd 2015, presented by healthcare region. The number of patients per region is presented within brackets

The analysis of treatment eligibility criteria includes all patients with at least one marker for disease stage (2672 patients, 78% of the cohort). Before and after July 2nd 2015, adherence to the treatment eligibility criteria was 80 and 87% respectively, with minor differences in adherence rates between the health care regions (Figs. 2 and 3).

**Fig. 4:**
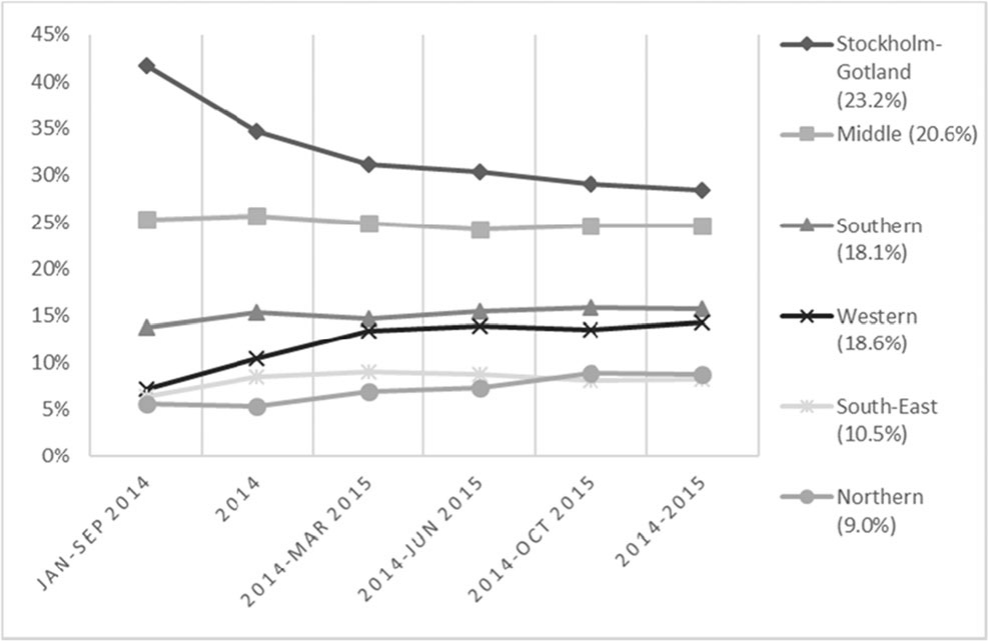
Distribution of treated patients by health care region over time. Percentages within brackets represent each region’s proportion of the general population as of June 30th 2015

Although the first protocol was implemented in November 2014 (Fig. 1), prescribing to patients started as soon as the first drugs were available. However, the rate of introduction varied initially between regions, and approached stable levels 15 months after the introduction of the drugs started (Fig. 4).

## Discussion

This cross-sectional study with national data on all patients selected for treatment with DAAs during 2014–2015 reflects the use of these drugs their first 2 years on the market, and covers a majority of the population initiated on treatment in Sweden during those years. It is not intended as a direct comparison between individual drugs, but to describe the patients treated and measure the extent to which the recommendations in the introduction protocol were implemented. Cure rates, based on data from 85% of the cohort, were generally as high as expected from either RCTs [17] or recent observational studies [18–23], with some variation between genotypes. Most of the patients were treated with the recommended combinations of the drugs under study, and the initially moderate adherence to the drug recommendations increased over time, with the increasing inclusion of drugs and drug combinations in the introduction protocol. Adherence to the treatment eligibility criteria was high and increased further when the restrictions were widened to also include patients with moderate liver disease. The rate of introduction varied between the regions, ranging between the more rapid uptake seen in the region of Stockholm-Gotland and the somewhat slower uptake seen in the western region.

The uptake of drugs among specialists is influenced by several factors [24] and other recent introductions in Sweden were less uniform and rapid, despite the implementation of a national introduction protocol [25]. The faster uptake of the DAAs in the region of Stockholm-Gotland, with approximately 25–30% of the Swedish CHC-population, is likely attributable to a larger number of prescribers with experience of using the drugs being introduced in earlier and ongoing clinical trials, and the fact that this region did not implement any further cost containment strategies than the introduction protocol. A recently published governmental investigation of the introduction process concluded that despite some initial regional variations in uptake of CHC treatment, the national introduction could be considered as uniform over the country [25].

Considering also the regional variations in other cost containment strategies and differences in tax base and patient populations between counties and regions, the results of this study demonstrate a considerable loyalty to the introduction process and protocol. The rate of adherence to treatment eligibility criteria was generally high, reflecting a high awareness among prescribers regarding the considerable costs and the need to restrict prescribing to those with the more severe stages of disease, even before the introduction protocols were implemented. Non-adherence may be related to occasional prescribing for unaccounted medical reasons or regional variations in disease severity among patients; this can however not be detected in the database. The initially moderate adherence to drug recommendations reflects the restricted number of drugs included in the protocol compared to the number of substances available on the market, but potentially also an initial variation in knowledge of the protocol itself. The legal status of the recommendations in the national introduction protocols—in relation to the existing legal framework—may also have been unclear to prescribers. To further legitimize the national introduction process and improve adherence to the future introduction protocols, this needs to be clarified [25].

Strengths of this study include a database with complete coverage on all patients’ prescribed/dispensed DAAs and clinical data on genotypes; stage of fibrosis and outcomes for almost 90% of the treated patients making is possible to analyze both drug utilization patterns and treatment outcomes. However, the study has several limitations. Even though the risk sharing agreements and the targeted governmental funding are considered important factors for this particular introduction [25], this study was not designed to determine the relative impact of the different, simultaneously implemented and rapidly changing policy measures on prescribing and uptake of the DAAs. Nor is it designed to detect and compare rates of undertreatment over time, i.e. the extent to which patients eligible for treatment based on disease severity are left without treatment. This would indeed have given a broader picture of the introduction rate than merely monitoring the relative proportion of patients initiated on treatment, but was not possible due to the limited amount of clinical data on untreated patients during 2014–2015. The definition of treatment eligibility is based on what can be reliably captured in either of the national registers used. Geographically varying completeness in InfCare Hepatitis and access to Fibroscan equipment for measurement of liver elasticity may have caused some patients to be misclassified as non-eligible, since their disease stage was not sufficiently documented or documented, but based on older liver biopsies. From July 2015, women with CHC preparing for in vitro fertilization were also considered eligible for treatment; this could however not be reliably captured and may thus have contributed to an underestimation of adherence to the treatment eligibility criteria. Patients treated as inpatients in hospitals were only included in the cohort if they were either registered in InfCare Hepatitis or had part of their drugs dispensed as prescription drugs. This may have influenced the patient population selected for this study as well as the estimated coverage of InfCare Hepatitis. However, the total drug volume of all patients completely or partially treated as inpatients, measured as packages sold over 2014–2015, is less than 2% of total volume [26]; hence, the impact of this methodological problem is likely to be minor.

## Conclusion

The conditions for introducing the first six of the second-generation direct-acting antivirals for chronic hepatitis C infection in Sweden were outlined in a national introduction protocol. The overall cure rate was estimated to 96%, with some variation between genotypes. Despite regional variation in other cost containment strategies, the high level of adherence to recommendations among prescribers and similar introduction rates in the regions indicate that the protocol contributed considerably to a rapid uptake and equal distribution of DAAs in Sweden.
